# Immunization with Lipopolysaccharide-Deficient Whole Cells Provides Protective Immunity in an Experimental Mouse Model of *Acinetobacter baumannii* Infection

**DOI:** 10.1371/journal.pone.0114410

**Published:** 2014-12-08

**Authors:** Meritxell García-Quintanilla, Marina R. Pulido, Jerónimo Pachón, Michael J. McConnell

**Affiliations:** Institute of Biomedicine of Sevilla (IBiS), University Hospital Virgen del Rocío/CSIC/University of Sevilla, Sevilla, Spain; The Scripps Research Institute and Sorrento Therapeutics, Inc., United States of America

## Abstract

The increasing clinical importance of infections caused by multidrug resistant *Acinetobacter baumannii* warrants the development of novel approaches for prevention and treatment. In this context, vaccination of certain patient populations may contribute to reducing the morbidity and mortality caused by this pathogen. Vaccines against Gram-negative bacteria based on inactivated bacterial cells are highly immunogenic and have been shown to produce protective immunity against a number of bacterial species. However, the high endotoxin levels present in these vaccines due to the presence of lipopolysaccharide complicates their use in human vaccination. In the present study, we used a laboratory-derived strain of *A. baumannii* that completely lacks lipopolysaccharide due to a mutation in the *lpxD* gene (IB010), one of the genes involved in the first steps of lipopolysaccharide biosynthesis, for vaccination. We demonstrate that IB010 has greatly reduced endotoxin content (<1.0 endotoxin unit/10^6^ cells) compared to wild type cells. Immunization with formalin inactivated IB010 produced a robust antibody response consisting of both IgG1 and IgG2c subtypes. Mice immunized with IB010 had significantly lower post-infection tissue bacterial loads and significantly lower serum levels of the pro-inflammatory cytokines IL-1β, TNF-α and IL-6 compared to control mice in a mouse model of disseminated *A. baumannii* infection. Importantly, immunized mice were protected from infection with the ATCC 19606 strain and an *A. baumannii* clinical isolate. These data suggest that immunization with inactivated *A. baumannii* whole cells deficient in lipopolysaccharide could serve as the basis for a vaccine for the prevention of infection caused by *A. baumannii*.

## Introduction

*Acinetobacter baumannii* is a Gram-negative coccobacillus with increasing clinical importance in the hospital setting. This organism is widely disseminated in the soil and water of natural environments [1], and can cause different types of infections as a nosocomial pathogen including pneumonia, bacteremia, meningitis and skin and soft tissue infection, among others [2]. This pathogen typically infects patients receiving mechanical ventilation and burn patients [3], however, it has also been isolated from community-acquired pneumonia samples [4], [5] and military personnel with traumatic injuries in Vietnam, Iraq, Kuwait and Afghanistan [6], [7]. Crude mortality rates associated with *A. baumannii* infection have been reported to be between 35% and 70% for nosocomial infections [8]. Importantly, due to the well-documented ability of *A. baumannii* to acquire antibiotic resistance, the number of multidrug and pandrug resistant strains has increased alarmingly in recent years [9], [10]. The global emergence of these highly resistant strains has severely complicated the clinical management of infections caused by *A. baumannii*. In this context of increasing antibiotic resistance, the development of an efficient vaccine against *A. baumannii* could contribute to reducing morbidity and mortality in certain patient populations [11].

The experimental vaccines that have been described for *A. baumannii* can be classified into two broad groups, vaccines that consist of a single purified antigen, and multicomponent vaccines. Within the first group, the outer membrane protein OmpA [12], the biofilm-associated protein Bap [13], the membrane transporter Ata [14], and the membrane associated polysaccharide poly-N-acetyl-β-(1–6)-glucosamine [15] have been reported as good candidates due to their ability to elicit specific immune response. However, survival experiments after active immunization have only been reported for OmpA, which showed partial protection, and Bap, whose expression in strains that do not form biofilms is unclear. The strategies employing multicomponent vaccines have included outer membrane complexes [16], outer membrane vesicles [17], and formalin-inactivated whole cells [18]. Each of these vaccines induced not only a potent immune response but also provided high levels of protection against *A. baumannii* infections in a murine model using both the ATCC 19606 type strain and clinical isolates. However, despite these promising results, the use of these multicomponent approaches in humans is complicated by the elevated endotoxin content of these vaccines due to the high levels of lipopolysaccharide (LPS) present in these preparations.

LPS consists of the O-antigen, a core polysaccharide and lipid A, the moiety responsible for the endotoxin activity of LPS. Early studies employing *Escherichia coli* demonstrated that the production of LPS was essential for bacterial viability [19]. However, it was later demonstrated that certain bacterial species, namely *Neisseria meningitidis* and *Moraxella catarrhalis*, were viable even after mutating the enzymes involved in LPS biosynthesis, resulting in a complete lack of LPS production [20], [21]. A recent report demonstrated that *A. baumannii* can acquire resistance to the peptide antibiotic colistin via mutation in the genes involved in the first steps of lipid A synthesis *lpxA*, *lpxC* and *lpxD* [22], resulting in strains completely deficient in LPS. These results indicate that *A. baumannii* is also viable in the absence of LPS production, raising the possibility that vaccines based on these LPS-deficient strains could be developed.

The objective of the present study was to develop an LPS-deficient inactivated whole cell (IWC) vaccine against *A. baumannii* and to characterize the immune response to immunization and its efficacy in a murine sepsis model. We demonstrate that the LPS deficient IWC produces a robust antibody response that is able to reduce post-infection tissue bacterial loads and provide protection against infection in a mouse model of *A. baumannii* infection.

## Materials and Methods

### Ethics Statement

All experiments involving the use of animals were approved by the University Hospital Virgen del Rocío Committee on Ethics and Experimentation (Evaluation code: 2013PI/296). In all experiments, efforts were made to minimize suffering, and any animals appearing moribund during the course of experimentation were immediately euthanized using thiopental.

### Bacterial strains

*A. baumannii* ATCC 19606 is an antibiotic susceptible reference strain. An LPS-deficient derivative of ATCC 19606 was obtained by plating an overnight culture of ATCC 19606 on Mueller Hinton agar containing 10 µg/ml of colistin, as described previously [22]. Strains with mutations in the genes involved in LPS biosynthesis were identified by sequencing the *lpxA*, *lpxC* and *lpxD* genes of the colistin resistant mutants that were present after overnight growth at 37°C. A strain with a large deletion in the *lpxD* gene was identified and designated IB010. Resistance to colistin was confirmed by broth microdilution according to Clinical Laboratory Standard Institute guidelines [23]. Absence of LPS was confirmed by measuring the endotoxin levels of three independent cultures of each strain using the QCL-1000 Limulus Amebocyte Assay (Lonza) according to the manufacturer's instructions. The Ab-154 strain is a previously characterized *A. baumannii* clinical isolate [24].

### Vaccine preparation and mouse immunization

The IWC vaccines (both LPS-containing and LPS-deficient) were prepared as described based on a previously described method [22]. Briefly, the ATCC 19606 and IB010 strains were grown in Mueller- Hinton broth to OD_600_ of 0.8. In the case of IB010, 10 µg/ml of colistin were added to the culture. In order to confirm the presence of the deletion after growth of IB010, three independent cultures of ATCC 19606 and IB010 were grown, and genomic DNA was isolated from each culture using the QIAmp DNA Mini Kit (Qiagen). The *lpxD* specific primers 5′ GCTAATTGGTGAAGGTAGTC 3′ and 5′ GACGAATCGTTTGAATCTGC 3′ were used to amplify genomic DNA from the cultures in order to confirm that the deletion in *lpxD* of IB010 was present after growth.

For vaccine preparation, bacteria were washed extensively in phosphate buffer saline before inactivation in 0.5 M formalin for 18 h with shaking at room temperature. Complete inactivation of the bacteria was confirmed by plating on blood agar. The concentration of inactivated cells was adjusted to 1×10^10^ cells/ml and combined 1∶1 (v/v) with the aluminium-based adjuvant, Alhydrogel 2% (w/v) (InvivoGen). Vaccination was carried out in 6 to 8-week-old, female C57BL/6 or BALB/c mice by intramuscular injection of 100 µl of the vaccine into each quadriceps muscle on days 0 and 14. Control mice were injected similarly with a mixture of phosphate buffer saline and adjuvant.

### Mouse model of *A. baumannii* infection

A mouse model of sepsis previously developed by our group and used for the evaluation of vaccines against *A. baumannii* was used to characterize the efficacy of the vaccine [25], [26]. This model produces a disseminated infection after intraperitoneal instillation of the inoculum, typically resulting in death within 24 to 48 hours. For preparation of the inocula, *A. baumannii* strains were grown for 18 h at 37°C in Mueller-Hinton broth cultures and adjusted to the appropriated concentration in physiological saline as described previously [8], [27]. Bacterial concentrations of the inocula were determined by plating on blood agar. Mice were infected on day 21 (one week after the second immunization) for C57BL/6 and on day 28 for BALB/c mice by intraperitoneal injection with 0.5 ml of the bacterial suspension and survival was monitored for 7 days.

### Spleen bacterial loads and serum cytokine levels

Post-infection bacterial loads were determined in vaccinated and control mice 12 h after infection. Mice were euthanized with an overdose of thiopental and after collection of blood samples from the retro-orbital sinus, spleens were aseptically removed, weighed and homogenized in 2 ml of physiological saline. Serial log dilutions were plated on blood agar plates for bacterial quantification. Serum levels of interleukin-1β (IL-1β), tumor necrosis factor alpha (TNF-α), and interleukin-6 (IL-6) were determined in mice at 12 h post-infection using BD OptEIA mouse kits (BD Biosciences).

### Enzyme-linked immunosorbent assays (ELISAs)

For indirect enzyme-linked immunosorbent assays (ELISAs), 96-well plates were coated with 5×10^7^ bacterial cells/well in phosphate buffer saline by incubating at 4°C overnight. ELISAs were performed using sera collected on days 0, 7 and 21 as described previously [28]. Antibody titers were measured against the strain which was used to immunize the mouse, and were defined as the dilution in which spectrophotometric readings were at least 0.1 units above background wells (wells containing no serum).

### Statistical analysis

Antibody titers, bacterial loads, and cytokine levels were compared using the Kruskal-Wallis H test and the Mann-Whitney U test for independent samples, and the Friedmann and Wilcoxon tests for dependent samples. The Bonferroni correction was applied when appropriate. Survival data were compared using the log-rank test. All statistics were performed using SPSS version 15.0 software (SPSS Inc.), and a p value of ≤0.05 was considered significant.

## Results

### Selection of an LPS-deficient strain for vaccine development

**Figure 1:**
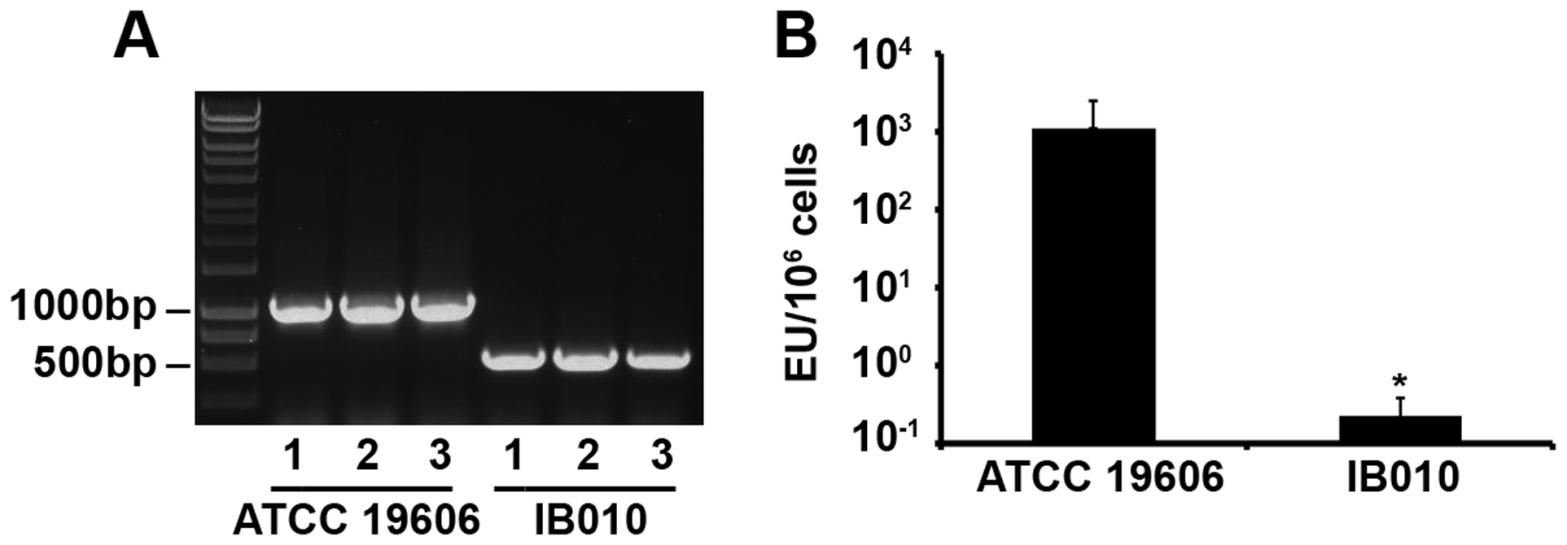
Mutation and endotoxin content of IB010. (A) Genomic DNA from three independent cultures of ATCC 19606 and IB010 was extracted and amplified using primers specific for the *lpxD* gene. The band corresponding to approximately 1000 Kb corresponds to the intact *lpxD* gene, whereas the faster migrating band corresponds to the *lpx*D gene with a deletion of 462 nucleotides. (B) Endotoxin levels of ATCC 19606 and IB010 determined by the Limulus Amebocyte Assay. Bars represent the median values of three independent cultures, and error bars represent the standard error of the mean. EU; endotoxin units.

Growth of ATCC 19606 in the presence of 10 µg/ml colistin resulted in numerous colistin-resistant derivatives with mutations in the *lpxA*, *lpxC* and *lpxD* genes (data not shown). One of these strains, IB010, contained a large deletion of 462 nucleotides in the *lpxD* (nucleotides 104–565) gene and was chosen for further use in vaccine studies. We reasoned that on the basis that the strain contained a large deletion, this strain would be less likely to revert to wild type during growth than strains containing single nucleotide changes or small deletions in the LPS biosynthesis genes. Broth microdilution experiments demonstrated that the minimum inhibitory concentration of the ATCC 19606 strain was ≤0.25 µg/ml and >128 µg/ml for IB010, demonstrating that, similar to results described previously [22], mutations in *lpxD* can result in resistance to colistin. In order to ensure that the IB010 was genetically stable during growth, genomic DNA from three independent cultures of ATCC 19606 and IB010 were amplified with *lpxD*-specific primers to confirm that the deletion was present. As shown in Figure 1A, a band corresponding to the mutated *lpxD* gene of IB010 containing a deletion of 462 nucleotides was present after amplification from three independent IB010 cultures. Phenotypic loss of LPS and reduction in endotoxin levels were characterized by the Limulus Amebocyte Assay for ATCC 19606 and IB010, and demonstrated that mutation in the *lpxD* gene resulted in a dramatic reduction in endotoxin levels to >1 EU per 10^6^ cells (Figure 1B).

### Antibody response to the LPS-deficient IWC vaccine

**Figure 2:**
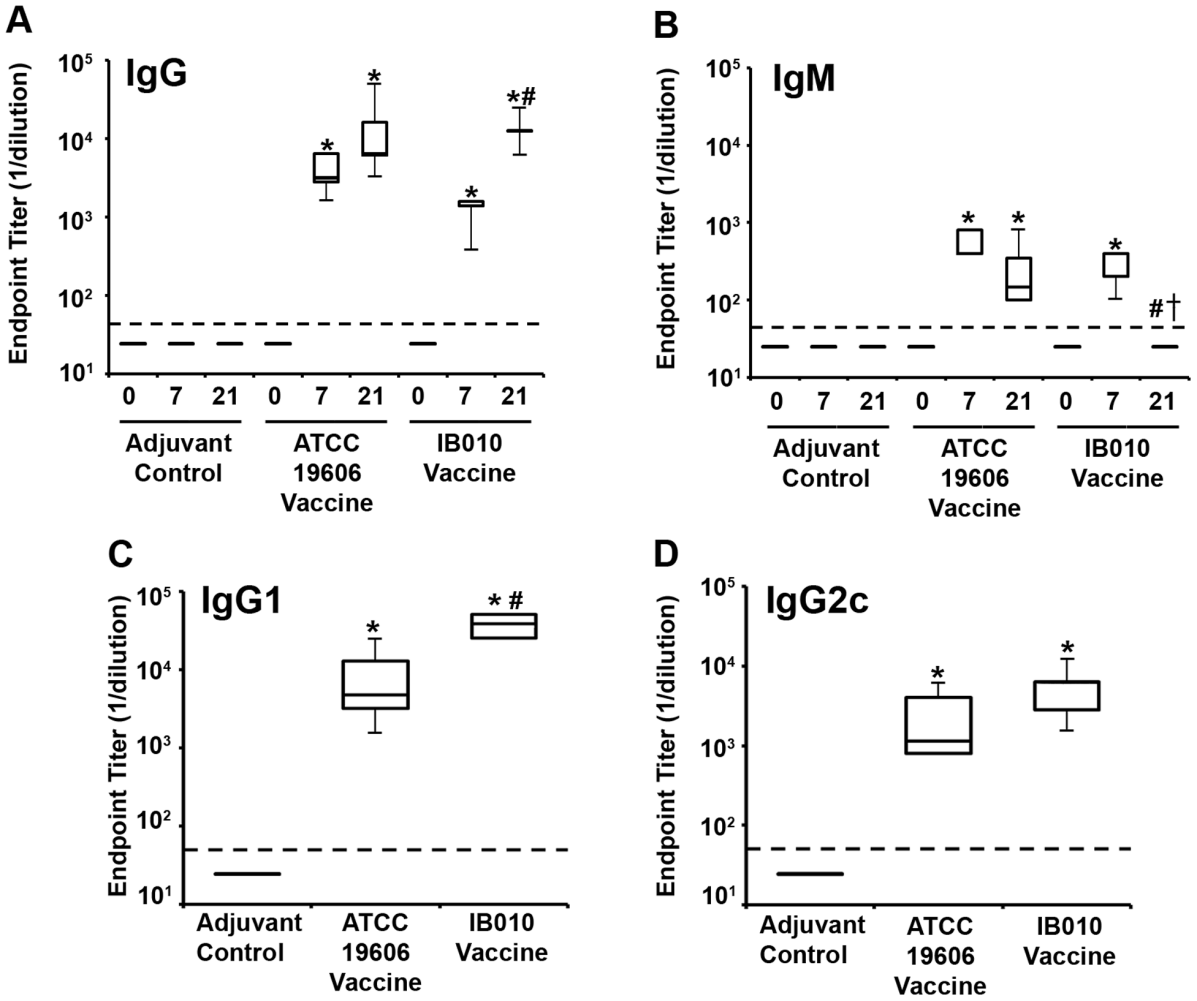
Antibody response to immunization with IB010. Serum samples were collected from ATCC 19606 vaccinated, IB010 vaccinated and control mice before vaccination (Day 0) and at day 7 and 21 after the first immunization, and levels of antigen specific total IgG (A) and IgM (B) were measured by ELISA (n = 8 mice/group). IgG1 (C) and IgG2c (D) levels in serum collected 7 days after the second immunization were determined in serum were measured by ELISA in ATCC 19606 vaccinated, IB010 vaccinated and control mice In all panels box and whisker plots represent the interquartile ranges and ranges, respectively, and horizontal lines represent median values. * p<0.05 compared to levels in control mice at the same time point, # p<0.05 compared to 7-day samples from the same experimental group, † p<0.05 compared to 21-day samples in ATCC 19606 vaccinated mice.

Formalin treatment of ATCC 19606 and IB010 resulted in no viable bacteria, indicating complete bacterial inactivation. No adverse effects were observed in mice vaccinated with inactivated IB010 and inactivated ATCC 19606 cells. In order to quantify the antibody response produced by immunization with inactivated IB010, indirect ELISAs were performed using sera collected from negative control mice (immunized with PBS and adjuvant) and mice vaccinated with 1×10^9^ inactivated IB010 cells. As a positive control, one group of mice was immunized with 1×10^9^ inactivated ATCC 19606 cells on the basis that we have previously shown that immunization with these cells induces a robust immune response and produces protective immunity against experimental infection [18]. As shown in Figure 2A, immunization with inactivated IB010 elicited detectable levels of antigen-specific total IgG in all mice seven days after a single intramuscular administration, and these antibody levels were significantly increased upon boosting with a second administration of the vaccine (p = 0.03 Wilcoxon test). Total IgG titers in mice receiving two administrations of inactivated IB010 vaccine were similar to titers in mice receiving the vaccine containing inactivated wild type cells (p = 0.726 Mann Whitney U test). Control mice had no detectable antigen-specific IgG at any point. In contrast, IgM levels were similar between mice immunized with the inactivated IB010 vaccine and mice receiving inactivated wild type cells seven days after a single administration (p = 0.186 Mann Whitney U test), however seven days after a second immunization there was no detectable antigen-specific IgM in IB010-vaccinated mice whereas all mice immunized with inactivated wild type cells had detectable levels of IgM (Figure 2B).

Levels of the IgG subtypes IgG1 and IgG2c, the IgG2a homolog in C57BL/6 [27], were determined in 21-day serum (Figure 2C and D). Both groups of mice receiving the inactivated vaccines had significant levels of IgG1 and IgG2c compared to control mice (p<0.001; Mann-Whitney U test). Interestingly, IgG1 titers were significantly higher in IB010-vaccinated mice compared to ATCC 19606-vaccinated mice (p = 0.003; Mann-Whitney U test), whereas IgG2c titers were similar between these groups. These results indicate that both Th1 and Th2 responses are elicited by the inactivated IB010 vaccine similar to what was previously shown for the inactivated ATCC 19606 vaccine [18].

### Effect of vaccination on post-infection bacterial loads

**Figure 3:**
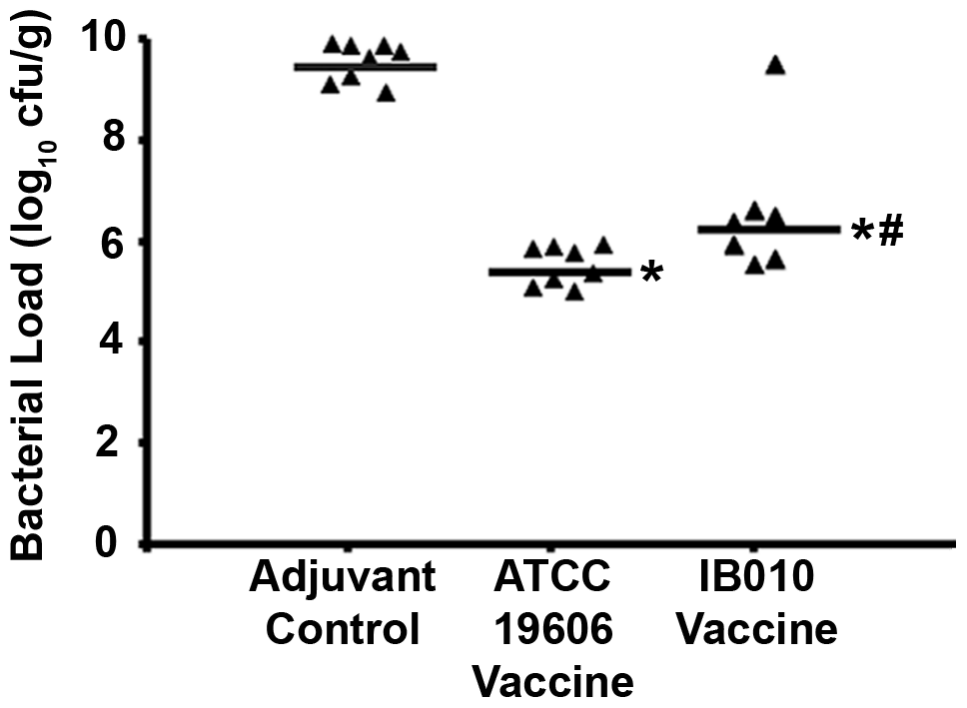
Effect of vaccination on tissue bacterial loads. Immunized and control mice were infected with 2.0×10^6^ cfu (300× LD_50_) of the ATCC 19606 strain and spleen bacterial loads were determined 12 hours post-infection (n = 8 mice/group). Data points represent bacterial loads from individual mice, and horizontal lines represent median values from groups of mice. * p<0.05 compared to control mice. # p<0.05 compared to ATCC 19606 vaccinated mice.

In order to characterize the effect of vaccination on post-infection tissue bacterial loads, we employed a mouse model previously developed by our group for the characterization of vaccine for preventing infection by *A. baumannii* [16]-[18]. This model rapidly produces a disseminated infection in which bacteria are detected in distal organs as soon as one hour post-infection [16]. Vaccinated and control mice were infected with 2.0×10^6^ cfu (300× LD_50_) of the ATCC 19606 strain, and 12 hours after infection spleen bacterial loads were determined (Figure 3). IB010 vaccination reduced the number of bacteria in spleens approximately 1000-fold compared to control mice (p<0.05; Mann-Whitney U test).

### Effect of vaccination on post-infection serum cytokine levels and survival

**Figure 4:**
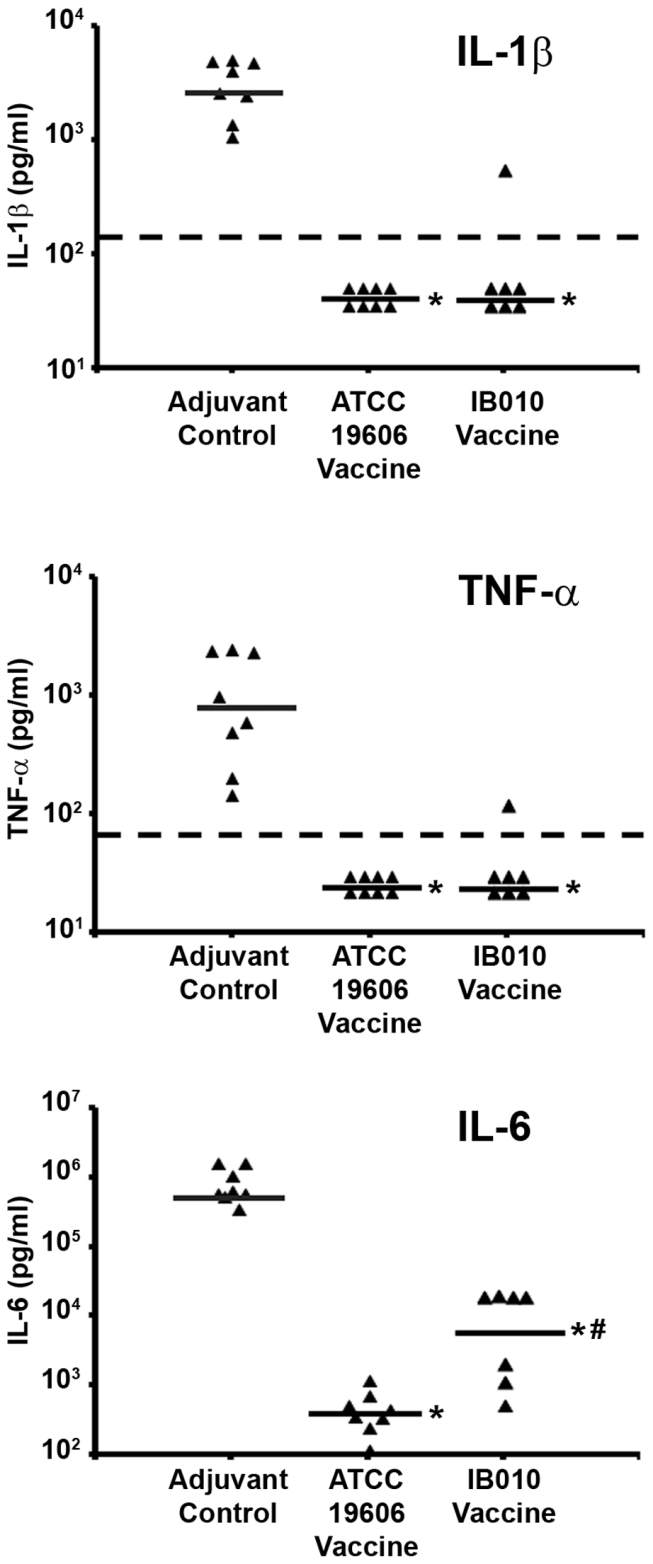
Effect of vaccination on post-infection pro-inflammatory cytokine levels. Immunized and control mice were infected with 2.0×10^6^ cfu (300× LD_50_) of the ATCC 19606 strain and serum levels of IL-1β, TNF-α, and IL-6 were determined (n = 8 mice/group). Data points represent cytokine levels from individual mice, and horizontal lines represent median values from groups of mice. * p<0.05 compared to control mice, # p<0.05 compared to ATCC 19606 vaccinated mice.

In order to characterize the effect of immunization with the inactivated LPS deficient vaccine on cytokine levels, sera were collected from vaccinated and control mice 12 h post-infection and the levels of IL-1β, IL-6 and TNF-α were determined (Figure 4). Levels of all three cytokines were significantly lower in both groups of vaccinated mice than in control mice (p = 0.003 for IL-1β, IL-6 and TNF-α; Mann-Whitney U test), suggesting that vaccinated mice did not experience the pro-inflammatory cytokine release associated with the development of septic shock.

**Figure 5:**
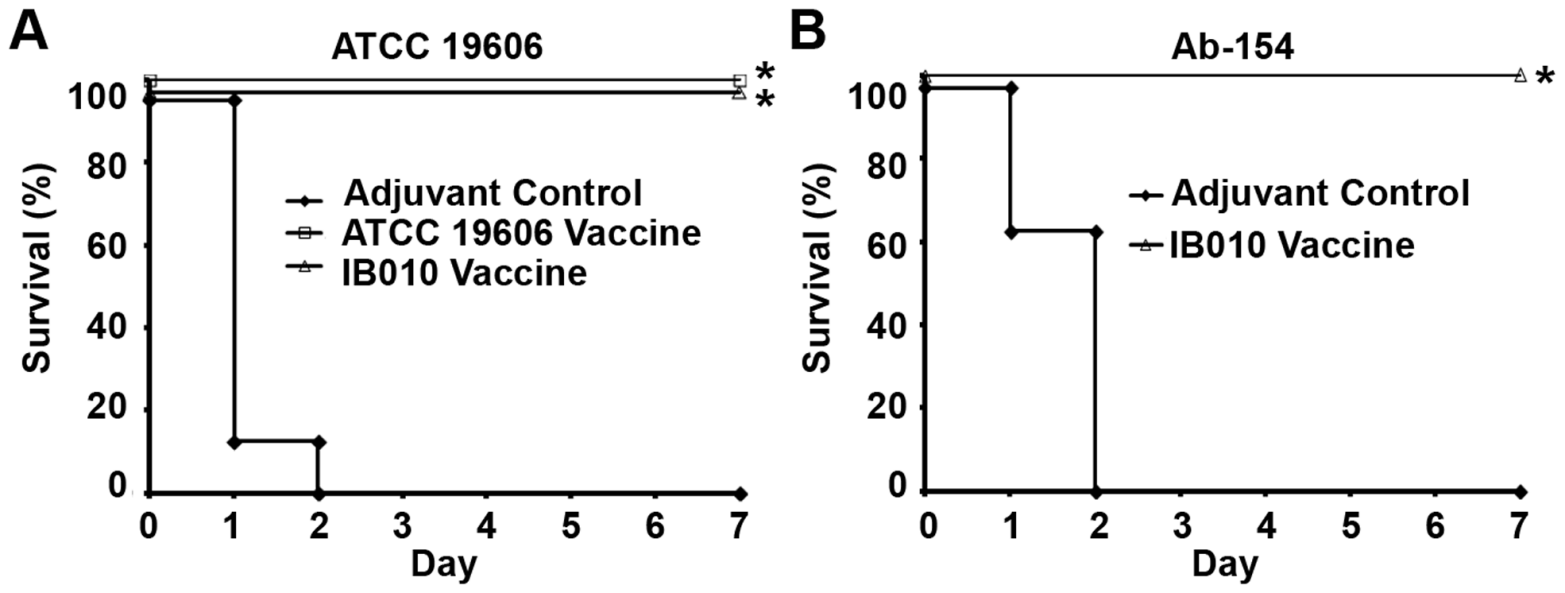
Effect of vaccination on survival in a mouse model of disseminated *A. baumannii* infection. Vaccinated and control mice were infected with 2.25×10^6^ cfu (340.9× LD_50_) of the ATCC 19606 strain (A) or 1.05×10^6^ cfu (2.18× LD_50_) of the *A. baumannii* clinical isolate Ab-154 (B), and survival was monitored over the following 7 days (n = 8 mice/group). * p<0.05 compared to control mice.

Vaccine efficacy was tested by infecting immunized and control mice with 2.25×10^6^ cfu (340.9× LD_50_) of the ATCC 19606 strain seven days after the second immunization, and survival was monitored over seven days (Figure 5). All mice vaccinated with the IB010 vaccine were protected from challenge, whereas all control mice died within 48 hours (P<0.001; log-rank test). As expected, all mice immunized with the ATCC 19606 strain survived challenge, similar to results that were previously reported [18]. In order to determine if vaccination with IB010 could protect against heterologous challenge with an unrelated strain, immunized and control mice were infected with 1.05×10^6^ cfu (2.18× LD_50_) of the previously characterized *A. baumannii* clinical isolate Ab-154 [29]. Once again, all immunized mice survived challenge whereas control mice succumbed to infection within 48 hours (p<0.001; log-rank test), indicating that immunization with IB010 can provide cross protection against challenge with a heterologous strain.

**Figure 6:**
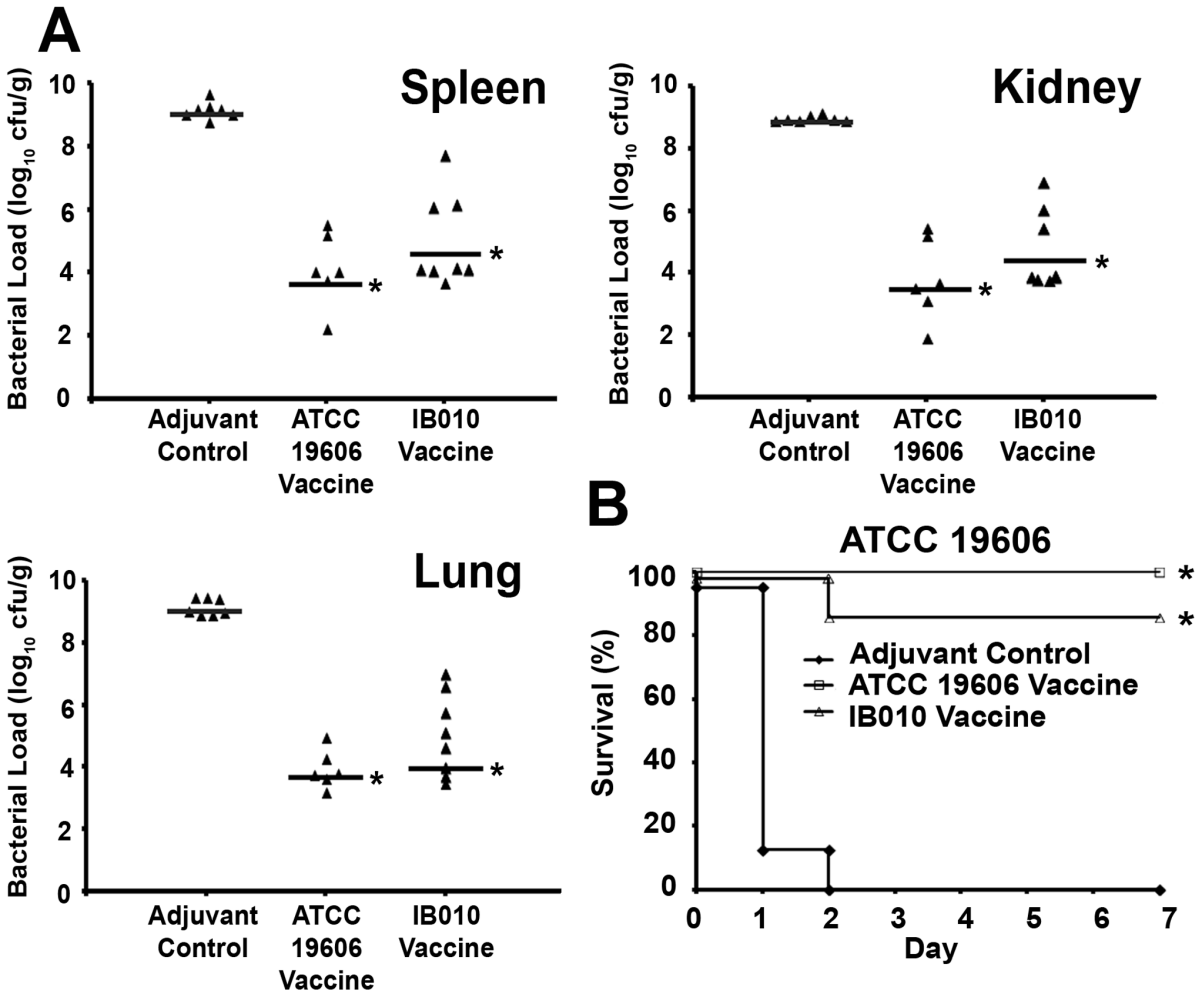
BALB/c bacterial loads and survival. Immunized and control mice were infected with 4.0×10^6^ cfu (4.14× LD_50_) of the ATCC 19606 strain. (A) Twelve hours post-infection spleen, kidney and lung bacterial loads were determined (n = 8 mice/IB010 vaccine group; n = 7 mice/control group; n = 6 mice/ATC 19606 vaccine group). Data points represent bacterial loads from individual mice, and horizontal lines represent median values from groups of mice. * p<0.05 compared to control mice. (B) Vaccinated and control mice were infected with 4.0×10^6^ cfu (4.14× LD_50_) of the ATCC 19606 strain and survival was monitored for seven days. (n = 7 mice/IB010 vaccine group; n = 8 mice/control group); n = 8 mice/ATC 19606 vaccine group) * p<0.05 compared to control mice.

We next wanted to characterize the immune response to vaccination and the protective capacity of the vaccine in a different mouse strain. As shown in Figure 6A, bacterial loads in spleens, kidneys, and lungs were significantly (approximately 1000-fold) lower in BALB/c mice vaccinated with the ATCC 19606 vaccine and the IB010 vaccine compared to control mice 12 hours after infection with 4.0×10^5^ cfu (4.14× LD_50_) of the ATCC 19606 strain (p<0.05; Mann-Whitney U test). There was no significant difference in bacterial loads between ATCC 19606 vaccinated and IB010 vaccinated mice. Survival experiments were carried out after inoculation with 4.0×10^5^ cfu (4.14× LD_50_) of the ATCC 19606 strain 14 days after the second immunization, and survival was monitored for seven days. Six out of seven mice immunized with the IB010 vaccine survived infection whereas all control mice succumbed to infection within 48 hours (p<0.001; log-rank test) (Figure 6B). These results indicate that the LPS-deficient vaccine shows similar characteristics in both C57BL/6 and BALB/c mice.

## Discussion

The development of a vaccine against *A. baumannii* for use in humans represents a novel approach for combating the increasing number of infections caused by multidrug and pandrug resistant strains. Although vaccines based on a single, highly purified bacterial antigen are attractive on the basis that these vaccine preparations are well defined and may produce a more predictable immune response, this approach has some limitations. Importantly, a vaccine targeting a single bacterial antigen would not have activity against strains that lack or down regulate the expression of the target antigen. Such a decrease in target antigen expression could occur due adaptation to immune pressure or during the acquisition of antibiotic resistance, since it has been shown that the expression of certain *A. baumannii* outer membrane proteins is decreased upon the acquisition of resistance [30]. Secondly, the process required for antigen purification may alter the conformation of the antigen such that the antibodies produced by immunization do not recognize the target antigen as efficiently. This is especially pertinent in the case of outer membrane proteins, which have commonly been used for the development of antibacterial vaccines including *A. baumannii* [12], [13], on the basis that the conformation of these proteins can be highly dependant upon their association with the bacterial membrane. Conversely, vaccines producing antibodies against multiple bacterial antigens may provide better coverage of circulating strains since the decreased expression of a single bacterial antigen would likely have less effect on the efficacy of such a vaccine. In addition, in the case of vaccines based on whole cells, the bacterial antigens are present in their native conformation.

Here we characterize the immunogenicity and efficacy of an IWC vaccine containing formalin inactivated cells of an LPS-deficient strain of *A. baumannii*. Importantly, this strain had extremely low levels of endotoxin activity and is genetically stable during growth due to a large deletion in *lpxD*. This is a crucial point given that one of the main problems associated with bacterial vaccines based on whole cells is the high level of endotoxin due to the presence of LPS. We demonstrate here that immunization with LPS-deficient whole cells produces a robust antibody response, in spite of the fact that LPS is known to have potent adjuvant-like activity, possibly related to its activation of toll-like receptor [31]. It has been shown that deficiency of LPS in *N. meningitidis* reduces the immunogenicity of outer membrane proteins in mouse models [21], [32], underscoring the adjuvant activity of LPS. In addition to the lack of the adjuvant effects of LPS, a second potential concern associated with the use of LPS deficient cells is that the conformation of the outer membrane antigens would be altered such that the antibodies produced by immunization would not be effective in neutralizing the infecting bacteria. Indeed it has been shown that *A. baumannii* strain lacking LPS have altered surface components in order to compensate for the lack of LPS [33]. The results presented here demonstrate that immunization with an LPS-deficient strain is capable of providing protection after infection with two LPS-containing strains, indicating that the elicited immune response is sufficient for recognizing antigens in their native conformation in LPS-containing strains.

To date, no survival studies have been reported in the literature using LPS-deficient strains as vaccines, however, the immunological response of LPS-deficient *Neisseria meningitides* and *Moraxella catarrrhalis* heat-killed whole cells has been addressed with poor and moderate immune response induction, respectively, compared to their wild type parental strains [20], [21]. The results obtained here are in contrast to the situation seen with *N. meningitidis*, in that immunization with an LPS-deficient strain of *A. baumannii* produces a robust humoral response based on IgG antibodies at levels similar to those elicited by immunization with the wild type strain containing LPS [21]. The reasons for these differences are, at present, unclear.

In conclusion, these results provide important information regarding the development of a vaccine for the prevention of infections caused by *A. baumannii* based on whole bacterial cells lacking LPS. These results may also provide insights into the possibility of developing vaccines for other bacterial species based on strains lacking LPS.
